# Responsible sharing of biomedical data and biospecimens via the “Automatable Discovery and Access Matrix” (ADA-M)

**DOI:** 10.1038/s41525-018-0057-4

**Published:** 2018-07-23

**Authors:** J. Patrick Woolley, Emily Kirby, Josh Leslie, Francis Jeanson, Moran N. Cabili, Gregory Rushton, James G. Hazard, Vagelis Ladas, Colin D. Veal, Spencer J. Gibson, Anne-Marie Tassé, Stephanie O. M. Dyke, Clara Gaff, Adrian Thorogood, Bartha Maria Knoppers, John Wilbanks, Anthony J. Brookes

**Affiliations:** 1 Harris Manchester College, University of Oxford, Mansfield Road, Oxford, OX1 3TD UK; 2 Public Population Project in Genomics and Society (P3G), McGill University and Genome Quebec Innovation Centre, 740 Dr Penfield Avenue, Suite 5104, Montreal, QC H3A 0G1 Canada; 3 Stewardly, Centre for Social Innovation, Suite 400, 215 Spadina Ave., Toronto, ON M5T 2C7 Canada; 4 IAMOPEN, Toronto, Canada; 5 Broad Institute of MIT and Harvard, 415 Main Street, Cambridge, MA 02142 USA; 6 CommonAccord, www.commonaccord.org, Paris, France; 7 Department of Genetics and Genome Biology, University of Leicester, Adrian Building, University Road, Leicester, LE1 7RH UK; 8 Centre of Genomics and Policy, McGill University, 740 Dr. Penfield Avenue, suite 5200, Montreal, QC H3A 0G1 Canada; 9 Walter and Eliza Hall Institute, 1G Royal Parade, Parkville, VIC 3052 Australia; 10 The University of Melbourne, Melbourne, VIC 3010 Australia; 11 Sage Bionetworks, 1100 Fairview Ave. N., Mailstop M1-C108, Seattle, WA 98109 USA

## Abstract

Given the data-rich nature of modern biomedical research, there is a pressing need for a systematic, structured, computer-readable way to capture, communicate, and manage sharing rules that apply to biomedical resources. This is essential for responsible recording, versioning, communication, querying, and actioning of resource sharing plans. However, lack of a common “information model” for rules and conditions that govern the sharing of materials, methods, software, data, and knowledge creates a fundamental barrier. Without this, it can be virtually impossible for Research Ethics Committees (RECs), Institutional Review Boards (IRBs), Data Access Committees (DACs), biobanks, and end users to confidently track, manage, and interpret applicable legal and ethical requirements. This raises costs and burdens of data stewardship and decreases efficient and responsible access to data, biospecimens, and other resources. To address this, the GA4GH and IRDiRC organizations sponsored the creation of the Automatable Discovery and Access Matrix (ADA-M, read simply as “Adam”). ADA-M is a comprehensive information model that provides the basis for producing structured metadata “Profiles” of regulatory conditions, thereby enabling efficient application of those conditions across regulatory spheres. Widespread use of ADA-M will aid researchers in globally searching and prescreening potential data and/or biospecimen resources for compatibility with their research plans in a responsible and efficient manner, increasing likelihood of timely DAC approvals while also significantly reducing time and effort DACs, RECs, and IRBs spend evaluating resource requests and research proposals. Extensive online documentation, software support, video guides, and an Application Programming Interface (API) for ADA-M have been made available.

## Introduction

Biomedical research is progressing rapidly, aided by new analytical technologies, “Big Data” strategies, and multi-disciplinary approaches. Studies are becoming ever larger in scale, and more detailed in nature, resulting in extensive resources (materials, methods, software, data, and knowledge) that need to be maximally shared, integrated, and exploited. Resource sharing is also increasingly encouraged by funders, who often mandate the creation of research sharing plans to encourage dissemination and re-use of resources generated through the use of public funds. This creates both challenges and opportunities for sharing so many diverse components in a timely manner, while creating as few barriers as possible for responsible research.

Resource sharing must abide by the legal and regulatory requirements and accepted good practices, including underlying constraints and considerations such as: specific consents provided by research subjects; laws and formal requirements set by local and other authorities; demands made by the resource creators who wish to protect themselves and avoid unsanctioned uses of the resource; intellectual property and commercialization plans; and technical and resource limitations relating to the actual process of sharing. Unfortunately, a rigorous approach to all of this is often lacking, in that sharing plans are often based on poorly formalized decisions, basic documents, generic contracts, and incomplete understandings. This lack of rigor in defining the terms and conditions for sharing is significantly exacerbated by the lack of a common “information model” for capturing and communicating these requirements^1^. This increases the costs, complexity, and burdens of resource stewardship, and decreases the efficiency of onward sharing, in terms of discovery and access of extant resources.

Consequently, not only are sharing rules and conditions often quite opaque to any one stakeholder when sharing is first initiated, but, as time passes and governance structures evolve, it becomes virtually impossible for groups such as Research Ethics Committees (RECs), Institutional Review Boards (IRBs), Data Access Committees (DACs), biobanks, data producers, and end users to confidently interpret and keep track of what is required and expected of all involved parties. This challenge becomes even greater as items are reused, widely shared, and recombined, potentially in automated settings.

In late 2015, members of the Global Alliance for Genomics and Health (GA4GH) and the International Rare Diseases Research Consortium (IRDiRC) organizations came together to discuss ways to improve resource sharing, especially biomedical data. This inspired the formation of the Automatable Discovery and Access (ADA) Task Team. The team comprised over 50 volunteer members from academia, industry, and the not-for-profit sectors, and was co-chaired by Anthony Brookes (University of Leicester) and John Wilbanks (Sage Bionetworks). The group worked together to create a much needed information model (a metadata standard) for consent and data use conditions. The product of this effort is the Automatable Discovery and Access Matrix (ADA-M, read simply as “Adam”), released online in late 2016 as a version 1.0 standard. Full support documentation is available online for this, along with support software, explanatory videos, and an Application Programming Interface (API) (for information on ADA-M in the contexts of the GA4GH’s broader regulatory and ethics efforts, see: https://www.ga4gh.org/ga4ghtoolkit/regulatoryandethics/. Accessed 30 May 2018. For information on ADA-M in the contexts of the IRDiRC’s efforts to advance knowledge on the natural history of rare diseases, see: http://www.irdirc.org/draft-version-of-ada-matrix-open-for-comments/. Accessed 30 May 2018. For a comprehensive description of how to use ADA-M, see the Guidance Document at: https://www.ga4gh.org/docs/ga4ghtoolkit/regulatoryandethics/ADAM_GuidanceDocument_15Dec2016_Final_v2.pdf. Accessed 30 May 2018. For a repository providing a reference API implementation of ADA-M, see: https://github.com/ga4gh/ADA-M. Accessed 30 May 2018. For videos on an overview of how ADA-M operates and on a more detailed user example, see: 10.6084/m9.figshare.6286982 and 10.6084/m9.figshare.6286985).

## ADA-M, an automatable discovery and access metadata standard

ADA-M was created to help increase the efficiency of resource discovery and access, by promoting responsible recording, versioning, communication, querying, and actioning of resource sharing plans. Datasets provide the clearest example for how resource sharing can benefit from the use of ADA-M, not least because the need to share data is so great and growing rapidly. The ADA-M metadata structure is flexible and can refer to any form of data asset, such as single data elements, individual records, full datasets, summarized forms of data, metadata, or even whole dataset collections and databases. For illustrative purposes we will focus primarily on data, yet it should not be forgotten that the ADA-M can equally benefit biospecimen resource sharing among biobanks and their users.

ADA-M’s objective is very straightforward. It seeks only to address the particular unmet need for a standardized format for regulatory metadata. It is not a project that aims to itself collect or store information about the conditions of use of data, nor does it try to influence whether or not any data should be collected or made available for sharing under any specific conditions. But by providing a consistent model for how to digitally structure the information about consent and other conditions of use regarding a given dataset, ADA-M will facilitate the responsible and efficient discovery and access of those data. The computer-readable metadata placed in ADA-M format in no way control or modify the data use requirements that pertain to data or other assets, but merely reflect them and make these requirements more accessible. Hence, the mandate of ADA-M is not to interfere in any way with the objectives of particular research projects, but only to help those projects function more efficiently in ICT-enabled contexts. In short, there remains a clear boundary between ADA-M’s purpose and the myriad objectives which drive the collection and use of biological data and resources.

By providing a practical structure for metadata by which any number of sharing conditions can be stated, making them unambiguous, computer-readable, and directly available for digital communication, searching, and automation activities, ADA-M fosters data sharing in accordance with the foundational principles of the GA4GH’s Human Rights-Based Framework for Responsible Sharing of Genomic and Health-Related Data^2^ (specifically Article 27, “Everyone has the right freely to participate in the cultural life of the community, to enjoy the arts and to share in scientific advancement and its benefits”. See http://www.un.org/en/universal-declaration-human-rights/index. Accessed 30 May 2018). To this end it is designed to be compatible with and work alongside other standardization efforts, software, and computing platforms, intended to help harmonize, rather than compete with, other initiatives.

An initial version of ADA-M was subjected to extensive alpha-testing over 4 months, by consultation with a wide range of individuals and groups. Collaborative cross-mapping exercises were also undertaken, using datasets managed by EGA, the Broad Institute’s DUOS system, a database of consent clauses, and Consent Codes^3^ (see Appendix 2 of the Guidance Document, “ADA-M v1.0 Structure” at: https://www.ga4gh.org/docs/ga4ghtoolkit/regulatoryandethics/ADAM_GuidanceDocument_15Dec2016_Final_v2.pdf. Accessed 30 May 2018). Based on feedback received, iterative rounds of improvement and retesting were undertaken. The task team proposed, debated, and democratically decided a series of progressive changes and improvements. The resulting version of ADA-M was released online in late 2016, since then it has been adopted in whole or in part by the Australian Genomics Health Alliance, the European Solve-RD project, members of the Health Data Research UK institute, and the US Broad Institute’s DUOS project, while in parallel it is being actively evaluated by the EU biobanking Infrastructure “BBMRI”, by Genomics England Ltd., by the Canadian Care4Rare-SOLVE project, and by the UK Tissue Directory and Coordination Center. Other expressions of interest have been received, leading ADA-M to recently be incorporated into several new funding proposals.

Such projects manage many and various datasets for a range of different purposes. Using the ADA-M model to structure regulatory metadata about their datasets will help them establish ICT-enabled ways to track and relay information about the conditions of use stipulated by, for instance, research subjects, journals, host institutes, biobanks, or granting agencies. These metadata, transformed into the ADA-M structure, constitute what we call an ADA-M “Profile”. How each project goes about interpreting source documents and collecting these elements that will make up the metadata, how they technically convert the regulatory information into a Profile, what granularity of information is placed in the Profile (even just a single use condition), whether this is done in real time by researchers or by a subsequent steward or consortia, and whether they wish to place the resulting (and possibly constantly evolving) Profiles into a centralized database or keep them alongside the referenced datasets, is all completely up to them to decide. Different approaches will emerge to suit different use cases, experiences will be shared, and new support tools and Profile representations will likely be developed to extend those we have already produced (see below).

## The core utility of ADA-M

ADA-M’s format facilitates responsible sharing of any type of biomedical resource in five key ways: first, it consolidates guidance on what might need to be addressed in a comprehensive resource sharing plan. Consolidation ensures creators of such plans, and RECs who advise on them, will no longer need to constantly improvise approaches that may be incomplete, diverse, and incompatible, and sometimes not truly fit-for-purpose. Second, it institutes a common method to capture and specify a resource sharing plan so that various facets of that plan are not easily lost, recalled incorrectly, or not versioned as situations change. Third, it provides a convenient means to properly communicate that plan so that those who need to enact, convey, or consult such a plan have a robust way to do so, and to do so within digital environments. Fourth, it enables detailed computer-based discovery (querying) of the sharing rules that apply to a given resource. This is especially helpful in federated architectures that necessarily need to employ interoperable metadata models. Computerized discovery helps to ensure time and effort is not wasted by DACs and potential users who discover resources, make requests, and evaluate requests only to eventually find that the proposed research is incompatible with one or more use conditions in the sharing plan. Fifth, it establishes a standard way to process resource access requests using scalable automated systems, or to connect and harmonize such systems, and thereby alleviate complete reliance on DACs, which are not easily scalable. Specifically, once an ADA-M Profile has been created for a given dataset, it allows the custodian to electronically and reliably check regulatory parameters within the Profile against requests made to access the data. It also enables a substantial reduction in ineligible requests to the DAC by filtering the results presented to researchers at the outset. This has the potential to greatly reduce administrative burdens on DACs and biobanks that implement ADA-M for their resource(s). This can be increasingly automated, bringing speed and increased precision to the process of assessing data sharing requests.

## The ADA-M metadata profile

The key to ADA-M’s utility lies in its ability to create tailored metadata “Profiles” (the data sharing rules populated into an ADA-M structure) for just about any set of regulatory conditions a given authority deems necessary. It is adaptable in terms of both the latitude and precision of the information it conveys, and yet it is structured to provide the consistency necessary for managing that information across regulatory spheres.

**Fig. 1:**
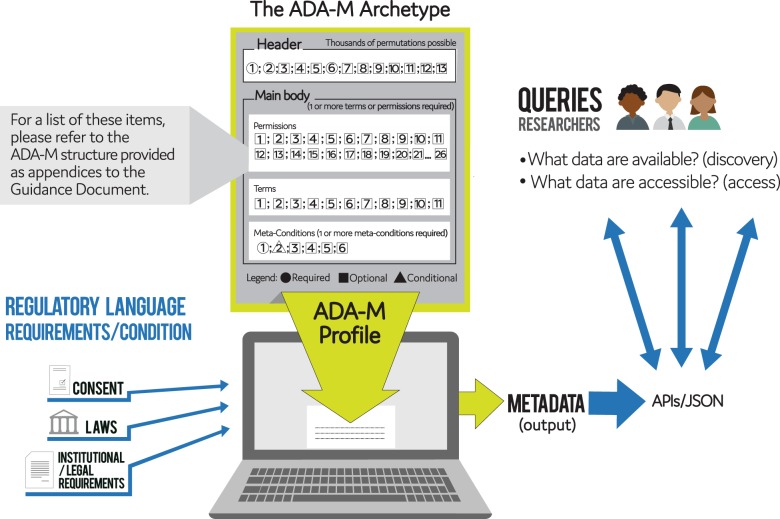
“The ADA-M Archetype”: The different combinations of numbers and shapes illustrate how an ADA-M metadata profile can accommodate many different permutations of regulatory factors and considerations, so that even highly complex information retains the standardized structure necessary for interoperable ICT contexts

**Fig. 2:**
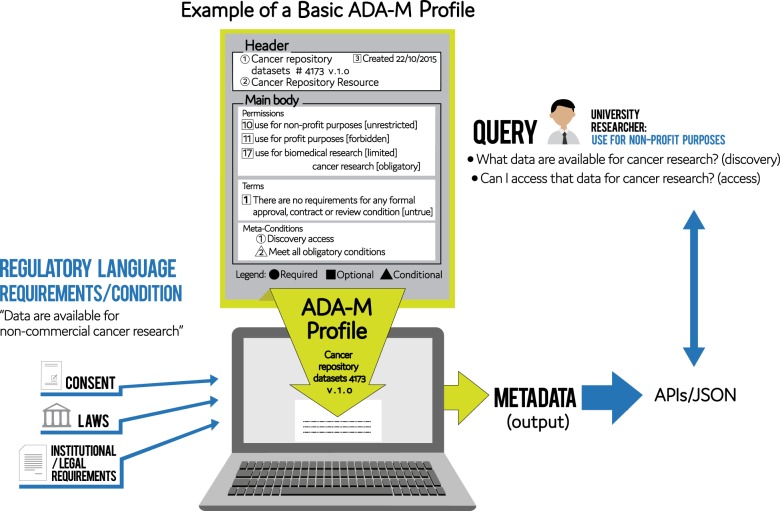
“Example of a Basic ADA-M Profile”: This illustration demonstrates how ADA-M creates a relatively basic metadata profile for a cancer research resource so the resource can be discovered by a suitable researcher. Whether simple or complex, the ADA-M Profile maintains the same standardized structure necessary for interoperable ICT contexts so such queries can be managed effectively

Profiles are created by entering applicable information into ADA-M’s series of hierarchically organized placeholders (see Figs. 1, 2). These are designed to separate out elemental data sharing concepts regarding conditions of use of a bioresource. These concepts cover a wide scope of considerations that could go into a data sharing plan. A user simply enters information into at least one of the placeholders to specify what is and/or is not permitted under the respective concept’s scope. Each of these inputs can be refined to whatever level of granularity at which the user wishes to operate. The format allows for a wide range of variables and permutations of concepts, while still maintaining a standardized overall structure.

An ADA-M Profile is not intended to directly recapitulate the exact and complete wording of limitations found, for instance, in consent forms or similar governance documents. Instead, users must understand and extract the critical aspects of the consents provided by subjects, and researcher or institutionally inspired conditions of use, to then place these in the appropriate place in the ADA-M’s structure. Aligning consent language, types of consent, and conditions of use to the ADA-M elements will often entail a process of interpretation of prior documentation, codifications, statements, and intentions. Interpretation of such matters has always been a critical and subjective step in data stewardship and oversight, essential for ensuring responsible use of data. It is not the goal of ADA-M to guide or control this step of interpreting the primary information, although we do anticipate that the widespread use of ADA-M would result in increased consistency in this function. ADA-M merely captures the final result in a clear, comprehensive, and standardized format which can then be used to ensure the referenced data are used responsibly and efficiently.

A completed ADA-M Profile could be anything from a very simple document comprising only the required Header sections and one Permission or Term concept entered (below), through to a highly detailed exposé of all aspects of a data sharing plan, including lots of free text explanations. It can therefore be made to suit requirements for different kinds of research projects, different types of researchers, and for research done in different regulatory regions. The Profile then allows data with specific use conditions to be efficiently matched up with appropriate research projects, enabling more fluid and effective sharing and availability of data.

## The structure of ADA-M

Full details of the ADA-M structure are provided online and a detailed Guidance Document is provided as Supplementary Data (see the Guidance Document at https://www.ga4gh.org/docs/ga4ghtoolkit/regulatoryandethics/ADAM_GuidanceDocument_15Dec2016_Final_v2.pdf). In brief, ADA-M v1.0 includes:

1. A HEADER section to capture contextual information about the ADA-M Profile itself and some basic statements about the data or other resource to which the Profile refers.

2. A MAIN BODY section that specifies and organizes regulatory concepts into 42 distinct categories that were felt to meet current needs (i.e., non-directive aspects of data use, that may or may not be employed to define acceptable use and conditions of use in any one setting), grouped into three sections, namely, “Permissions” (mainly relevant to consent), “Terms” (typically relating to legal/contractual matters), and “Meta-Conditions” (over-arching topics).

The HEADER section is completed only once within a single ADA-M Profile. It comprises 13 items, 3 of which are required (i.e., values must be provided) whereas the other 10 are all optional (may be left empty). The MAIN BODY section is completed one or more times within a single ADA-M Profile, with multiple copies being needed when more than one different combination of data use criteria have to be stated, or when different data use criteria are to apply for discovery versus access. In such situations the different completed copies of the Main Body are entered sequentially in the structure of the completed Profile document, all under one Header section. The MAIN BODY comprises 42 concepts, only one of which is a required field. All other fields are optional, so long as at least one of the main fields in the “Permissions” or the “Terms” sections are given a value (i.e., so the Profile asserts at least one condition of use). All fields without values can be left blank and not reported in the final ADA-M Profile.

The MAIN BODY also enables conditionalities and obligations to be specified. Conditionalities arise when a particular use permission (e.g., use for research on disease X is allowed) is conditional on some other part of the Profile (e.g., resulting data must be made public). Obligations involve situations where a particular use is not only permitted to occur but is obligated to occur (e.g., use for research on disease X is a necessity).

The MAIN BODY comprises three sub-sections:

### A permissions section

A permissions section covers 26 hierarchically arranged concepts generally relating to laws, institutional policies (Data/Sample Access Policies, Material/Data Transfer Agreement, Data Access Agreement, etc.), and consents. For these concepts a single value is entered from a list of permitted options. Permitted values include “Unrestricted” and “Limited” which are available for all the concepts, as well as “Unrestricted[Obligatory]”, “Limited[Obligatory]”, and “Forbidden” which are additional options for 23 of the 26 items. The latter three options are not available for three concepts of use which must unavoidably occur in some manner or another (“use within countries/locations”, “use by organizations”, “use by categories of person”) and so these cannot be logically “Forbidden” or made “Obligatory”. Each entered value can optionally also be supported by a free-text statement in an accompanying field provided for this purpose. This allows for further elaboration of any condition of use, to be read by human users of the Profile. More specifically, the free-text fields are used: to specify what forms of use are allowed, are not allowed, or must occur; to elaborate on why a type of use is unrestricted, forbidden, or obligatory; and to state conditionalities of use. For example, when “Limited” or “Limited[Obligatory]” is entered it will normally be useful to provide further textual details, ideally as a list of permitted or prohibited options (e.g., “Use permitted for research on diseases A, B, and C. Use prohibited for research on diseases X, Y, and Z”). Items on such a permissions list that are obligatory are indicated by adding the “[Obligatory]” suffix to that item in the list, e.g., “Use permitted for research on diseases A, B[Obligatory], and C”—meaning the research use must relate to disease B, and optionally also to diseases A and/or C.

### A terms section

A terms section covers 11 different areas of formalized governance and behavior that are generally stipulated by laws, regulations, institutional policies, researchers, or ethics oversight bodies, as a condition of granting access to, or continued use of, a given set of data. These considerations may be useful to know about in a discovery context, but assist particularly when actually seeking access.

Terms concepts are each very broad, aiming to together cover all possible considerations, and are not hierarchically arranged. Each concept is optional, and can accept one of two possible values (“True” or “Untrue”), and has an associated free-text field to be read by human users. One category (“There is no possibility of recontacting data subjects”) has two free-text fields, designed to separately capture details on subject recontacts which may or which must occur, respectively. Terms concepts are expressed as “negatives”, e.g., “There are no requirements regarding collaboration”. Therefore, entering the value “True” conveys the fact that no collaboration with the data provider is required. Conversely, the value “Untrue” means that some policy or condition relating to collaboration does apply, the details of which will typically (though optionally) be elaborated in the accompanying free-text field, which could include a URI (HTTP hyperlink) to a document containing further details about such policies or conditions. Free-text fields may also be used to state conditionalities that apply to any Terms concept.

### A meta-conditions section

A meta-conditions section comprises five concepts that sit outside the respective realms of the Permissions and Terms sections. It addresses five very specific matters, and so each concept is designed to be filled in with only one value from its own particular list of permitted values. Only one of these concepts is required, namely, “Mode of sharing”, which specifies the nature of the sharing activities that the Profile concerns (i.e., Discovery and/or Access). Three Meta-Conditions concepts are optional, and one is conditional.

(“Interpretation rule if multiple Obligatory permissions are specified” also being optional unless two or more entries in the Permissions and Terms sections are stated to be “[Obligatory]” in which case this field must be filled in.) Only one Meta-Conditions concept (“There are no other use restrictions/limitations in force which are not herein specified”) has an accompanying free-text field to optionally provide further details.

## Computing, validation rules, and the broader compatibility of ADA-M Profiles

When we take into account all the possible permutations of the categories and subcategories available in ADA-M, tens of thousands of unique metadata Profiles can be created, each of which describes different usage parameters. Yet the structure by which this information is recorded remains fixed and interoperable, allowing it to be used by a wide variety of computing platforms and software, and to work (and evolve, as need be) alongside other standardization efforts.

### Computing

Three envisioned basic uses of ADA-M involve computer handling of completed Profiles. First, ADA-M is a means for unambiguously communicating data use conditions from one system to another, for interpretation or display. There are two standard formats currently available for such transfers, a JSON document and a key-value pair text document (see Appendix 4 of the Guidance Document, “Document Formats for ADA-M v1.0 Profile” at https://www.ga4gh.org/docs/ga4ghtoolkit/regulatoryandethics/ADAM_GuidanceDocument_15Dec2016_Final_v2.pdf). Second, ADA-M is a means for enabling discovery services to include data use conditions in the range of characteristics they permit a user to query. Beyond adhering to the core syntax, semantics, and validation principles of ADA-M, it remains a matter of local choice what document or databasing format and technology is used for such applications. Third, ADA-M is a means for automated decision making on granting access to data. Given the complexity of some usage requests, and of some use condition Profiles, and also considering the normative issues surrounding some social groups as well as the many unknowns entailed in Big Data analytics,^4^ it is unlikely that this task will ever be fully automated. However, in cases where a decision engine merely has to formulaically evaluate straightforward Permissions values, and Terms values, and no vulnerable groups are involved, then such adjudications might be left to the computer so as to partially relieve the growing financial and personnel burdens being placed on DACs. At a minimum, ADAM can reduce the triage burden on DAC and Biobank administrators, so that they need only process and give consideration to eligible access requests.

### Validation rules

As the complexity of a Profile increases, so does the potential of having internal inconsistencies, or anomalous entries (such as a completed text field under a Permissions concept for which no main value has been entered). To prevent this, a set of seven validation rules has been defined that address the problems that are most likely to occur (see Appendix 3 of the Guidance Document, “Rules for Completing a Valid ADA-M, v1.0 Profile” at https://www.ga4gh.org/docs/ga4ghtoolkit/regulatoryandethics/ADAM_GuidanceDocument_15Dec2016_Final_v2.pdf). These have been implemented as automated checks within a dedicated software module that assists users in completing ADA-M Profiles (see https://www.ga4gh.org/ga4ghtoolkit/regulatoryandethics/ or http://www.irdirc.org/draft-version-of-ada-matrix-open-for-comments/. Accessed 30 May 2018).

### Broader compatibility

ADA-M is designed to be compatible with and work alongside other standardization efforts that cover different aspects of digitization and automation regarding consent and data use conditions, including the Oasis eXtensible Access Control Markup Language,^5^ the HL7 Healthcare Privacy and Security Classification System,^6^ the FHIR Consent Directives,^7^ the Consent Codes specification (for those specific codes, see https://www.ga4gh.org/docs/ga4ghtoolkit/regulatoryandethics/DataUseBeacon_160209_tab_0.pdf. Accessed 30 May 2018), the Genetic Alliance Platform for Engaging Everyone Responsibly,^8^ and the Broad Data Use Oversight System (DUOS).^9^ All such initiatives have a certain degree of overlap regarding the actual terminologies used and their definitions, and the ADA-M developers are pleased to be working with a Data Use Ontology^10^ project led from the European Bioinformatics Institute to formulate a common ontology for this domain.

## Conclusion

Clearly, it will take much work on many fronts to fully optimize biomedical data and biospecimen sharing. Capture and organization of heterogeneous, yet crucial, regulatory information is an important first step. ADA-M can act as a catalyst that quickens the process by enabling this information to be placed in a standardized and interoperable structure.

ADA-M is designed to expand, evolve, and adapt with use. Part of the input for ADA-M’s ongoing development will come from considering the types of information users enter into the free-text fields mentioned above. Options for ontologies that might be employed to bring more standardization to this aspect of the metadata model, and how to fit them into the model, are already being deliberated. The ongoing development of support software and the ADA-M reference API will continue, and other valuable areas of development are being considered, such as defining ways to reliably associate ADA-M Profiles with the data entities to which they refer, and creating a standardized data request structure that will correspond to ADA-M.

ADA-M is now ready for use in many settings. Potential adopters of ADA-M and expert volunteers who might want to contribute to the project are warmly encouraged to signal their interest and join the team.

### Data availability

No datasets were generated or analyzed during the current study.
